# Asian Australian Experiences of Racism During the COVID-19 Pandemic in Victoria: a Preliminary Analysis

**DOI:** 10.1007/s12134-023-01018-8

**Published:** 2023-03-01

**Authors:** Glenda Ballantyne, Vincent Giarrusso

**Affiliations:** School of Social Sciences, Media, Film, and Education, Swinburne University of Technology, John St, Hawthorn, 3122 Australia

**Keywords:** Anti-Asian racism, Racisms, COVID-19 pandemic, Australia

## Abstract

Since the outbreak of COVID-19, reports of racism and racial discrimination towards those of an Asian cultural background have increased in culturally diverse countries around the world. The current study sought to gain clarity about Asian Australian experiences of racism by conducting inferential and descriptive analysis of cross-sectional survey data collected from the Australian state of Victoria (*n* = 436). Building on previous studies which have identified a range of modalities and consequences of COVID-19-related racism, participants were prompted to consider their experiences of racism in the year prior to the outbreak of COVID-19 as well as their experiences during the pandemic on four measures—Direct Experiences of Racism, Vicarious Experiences of Racism (online and in-person), Everyday Racism and Hypervigilance. For the target group (participants with an East or Southeast Asian cultural background resident in Victoria), analysis showed an increase in experiences across three of the four measures, with small to moderate effect sizes, Everyday Racism (*r* = 0.22), Vicarious Experiences of Racism (*r* = 0.19) and Hypervigilance (*r* = 0.43). Analysis demonstrated that the target group experienced a significant increase in online experiences of racism (*r* = 0.28). These findings shed light on the contradictory findings of previous research on pandemic-related racism in Australia. We show that the pandemic impacted Victorians likely to be perceived as of Chinese background more than other Asian Australians.

## Introduction

The COVID-19 pandemic has created major disruptions in societies around the world, disturbing the patterns of everyday life in multiple and often dramatic ways. However, both anecdotal reports and systematic studies suggest that the impact of the pandemic has varied widely across countries and among different communities within them (Delvin & Connaughton, 2020). A stark example has been the disproportionate impact on minoritised communities in culturally diverse societies. Research has shown that minority groups have borne a double burden of economic vulnerability and disproportionate exposure to the health threat of the pandemic (Elias et al., 2021; Gravlee, 2020; Mansouri, 2020). The pandemic has both exposed and exacerbated existing structural discrimination and social inequities. At the same time, researchers have also pointed to the emergence of a ‘second’ or ‘shadow’ epidemic of pandemic-related interpersonal racism (Godlee, 2020). This pandemic has targeted multiple marginalised communities (Doery et al., 2020, 2022; Devakumar et al., 2020; Godlee, 2020; Mansouri, 2020). In particular, the origin of the COVID-19 virus in China - and its politicisation by the Trump administration—has seen a ‘re-emergence of a form of politicized ethno-cultural racism’ (Elias et al., 2021, p. 784) aimed specifically at people with Asian backgrounds. The Centre for the Study of Hate and Extremism (2021, p. 3) reported an ‘historic surge in anti-Asian hate crime’ in the USA as a consequence of the COVID-19 pandemic, while the European Union Agency for Fundamental Rights concluded that the ‘coronavirus pandemic triggered an increase in racist and xenophobic incidents against people of (perceived) Chinese or Asian origin’ (2020, p. 11).

Reports of anti-Asian racism were also widespread in the early phase of the pandemic in Australia, where globally circulating discourses of the ‘China virus’ and the ‘Kung-flu’ were intensified by deteriorating relations between China and Australia over geopolitical and trade issues (Razik, 2020; Hurst, 2021; Zhou, 2021; Tan, 2020; Asian Australian Alliance 2021). Alongside these reports of pandemic-related incidents of racism, both NGOs and scholars found evidence of disproportionately increased levels of stress and anxiety among Asian Australians (Doery et al., 2020; Biddle et al., 2020; Kassam & Hsu, 2021).

Early Australian studies reported more mixed findings concerning the scale and dynamics of the shadow pandemic than their international counterparts. While many studies reported multiple incidents of pandemic-related racism and discrimination experienced by Asian Australians (Farbenblum & Berg, 2020; Nguyen & Balakrishnan, 2020; Gallagher et al., 2020; Weng et al., 2021; Doery et al., 2020; Kassam & Hsu 2021; Kamp et al. 2022), several large-scale surveys found no increase—and sometimes a decrease—in racism and discrimination experienced by Asian Australians as a result of the pandemic (Biddle et al., 2020; Kamp et al., 2022).

This paper seeks to shed light on these contradictory findings by more specifically examining the contexts, modalities and targets of pandemic-related racism in Australia. Drawing on data from an online survey of 436 Asian Australians residing in Victoria conducted between 3 May 2021 and 7 June 2021, we seek to better understand the variations in how the shadow pandemic has unfolded in Australia. First, we focus on a single state jurisdiction. Different levels of infection and divergent public health responses played out in markedly different scenarios across Australia’s eight states and territories (Parliament of Australia, 2020; Australian Institute of Health and Welfare, 2021). It is therefore possible that levels of racism and discrimination have varied across state jurisdictions, and that national studies obscured the impact of the pandemic in the most hard-hit states. While our focus on Victoria, which was subjected to the most stringent restrictions in the country, cannot provide comparative data, it offers insight into a jurisdiction subject to high levels of stress.

Second, we differentiate between subcategories of ‘Asian Australians’. Although previous research recognised that people likely to be perceived as having Chinese heritage experienced higher levels of racism than other Asian Australians (Biddle et al., 2020; Kamp et al., 2022), it predominantly analysed data for the broader group of ‘Asian Australians’. As a result, it is possible that these studies diluted the impact of the pandemic on those most likely to be targeted. To examine this, we compare the experience of the whole sample with that of those who are likely to be perceived as of Chinese background.

Finally, our analysis distinguishes between three modalities of racism—direct experiences, vicarious experiences (both in-person and online) everyday racism which the pandemic differentially constrained and enabled. Earlier studies did not consider all of these.

We address two research questions:

1. Did people who were likely to be perceived as Chinese experience an increase in racism compared to other Asian Australians in Victoria during the pandemic?
2. What were the predominant modalities in which this racism was experienced?

## Background—the Impact of the COVID-19 Pandemic on Asian Australians

Australia’s experience of the pandemic was shaped by two federal government decisions. The first was to act early to close the country’s international borders (Stobart & Duckett, 2021). The second, arguably a non-decision, was to delay ordering of vaccines longer than many comparable countries (National Centre for Immunisation Research and Surveillance, 2022). Following from these decisions, an ‘elimination’ strategy was maintained longer than in many comparable countries. Within this national framework, however, the experience of the country’s six states and two territories, which held jurisdiction over health responses, varied in relation to both severity of outbreaks and political and public health responses. Among them, the Victorian experience stood out. One of the two most populous and racially and culturally diverse states in Australia, it experienced high levels of COVID-19 and longer lockdowns than any other jurisdiction, with its capital, Melbourne, earning notoriety as the world’s most locked down city (Tuffield, 2021).

Widespread media reports of a spike in racism and discrimination early in the pandemic prompted a number of Australian studies to examine its impact on minoritised groups. The plight of international students, who were given little support from the federal government, was a prominent focus (Farbenblum & Berg, 2020; Gallagher et al., 2020; Weng et al., 2021). There were also several attempts to provide a more holistic picture. One was a ‘rapid response’ in-house survey of 376 young people of diverse cultural backgrounds in Victoria conducted by the Centre for Multicultural Youth and the Australian National University in June 2020 (Doery et al., 2020). Seeking to understand the impact of the shadow pandemic (Doery et al., 2020, p. 5) on the social and emotional wellbeing of young Victorians, the study compared ‘direct’ and ‘vicarious’ (witnessing or hearing about others’ online) experiences of Anglo/European and non-Anglo/European background participants. It found a consistent disparity between the two groups. For example, 32% of non-Anglo/European participants compared with 8% of Anglo/Europeans had experienced more than six incidents of direct racism or discrimination during the pandemic. In relation to vicarious online racism, 79% of non-Anglo/European compared with 42% of Anglo/European background participants reported 3–4 experiences of discrimination (Doery et al., 2020, p. 20). The study also found a marked disparity in ‘experiences of feeling anxious in anticipation of experiencing racially motivated hostility’, with 69% of the non-Anglo/European group compared with 41% of Anglo/European group reporting 3–4 such experiences during the surveyed period (Doery et al., 2020). The disparity between the two groups was even greater in relation to anxiety due to anticipating ‘vicarious’ exposure such as seeing racism online or in the media (79% compared with 42%) (Doery et al., 2020, pp. 8–9).

A different picture of levels of racism and discrimination during the pandemic emerged from a larger, national survey of 3043 Australians, of whom 334 self-identified as Asian Australians, conducted in October 2020 as part of an ongoing longitudinal study (Biddle et al., 2020). This study reported a large discrepancy in levels of stress and anxiety among Asian Australians compared with the whole population. It found that 80.7% of Asian-Australians reported being anxious and worried due to COVID-19 compared with 62.4% of the rest of the Australian population (Biddle et al., 2020, pii). However, looking at discrimination in face-to-face settings, it found that there had been no increase in experiences of racial discrimination. Coupled with their finding that Asian Australians had fared worse in economic terms during the COVID-19 pandemic compared with the rest of the Australian population, the authors concluded that the increased levels of anxiety were more likely related to economic outcomes (Biddle et al., 2020).

A similar conclusion about levels of racism emerged from another large-scale, national survey of 2003 self-identified Asian Australians conducted between November 2020 and February 2021 (Kamp et al., 2022). Seeking to gauge the type and frequency of Asian Australians’ experiences of both face-to-face and online racism during the pandemic, as well as the impact on mental health, it found that racism occurred most frequently online and that in this setting, racism was predominantly connected to the pandemic (Kamp et al., 2022, pp. 4–5). It also documented the mental health and wellbeing consequences, finding that the anticipation of racism was felt by the majority (71.5%) of participants who had experienced online racism during the pandemic, and 81.2% avoided specific situations because of the potential of encountering racism (Kamp et al., 2022, p. 16). It also found that such racism was eroding the ‘resilience’ of Asian Australians, reporting an almost 15% increase in participants who found it difficult to ‘snap back’ during the pandemic associated with experience of online racism. Overall, however, it found that there was a decrease in racism experienced by Asian Australians online and more broadly (Kamp et al., 2022).

Two annual surveys conducted by the Lowy Institute provide some of the few sources of data on the experience of Chinese Australians. The first was conducted in November 2020 during the first year of the pandemic (Kassam & Hsu, 2021). The second was conducted the following year. The 2020 survey found that a significant minority of participants had experienced threats, attacks and discriminatory treatment because of their Chinese heritage. Among the 1040 respondents, 31% reported that they had been called offensive names and 37% that they had been treated differently or less favourably because of their Chinese heritage (Kassam & Hsu, 2021, p. 17). The survey conducted at the end of 2021 found that Chinese Australians continued to face discrimination and negative treatment in Australia, with one in three respondents reporting having been treated differently or less favourably in 2021 because of their Chinese heritage (Kassam & Hsu, 2022, p. 2). Around 34% said they have been treated ‘differently or less favourably because [they] are of Chinese heritage’, a marginal decrease from 37% in 2020. Fewer Chinese Australians reported verbal abuse, with 25% saying they ‘have been called offensive names because [they] are of Chinese heritage’, down six points from 2020. However, the proportion of respondents (33%) who said someone ‘expressed support for [them] because of their Chinese heritage’, declined by seven points from 2020.

These studies each shed light on aspects of the impact of racism in Australia related to the COVID-19 pandemic. However, elements of their survey designs have the potential to obscure nuances in the scale and impact of pandemic-related racism. National studies offer broader reach, but they miss variations across different jurisdictions, especially around differences in duration of lockdowns which in turn influenced opportunities for interactions in face-to-face settings where incidents of racism often occur. Studies reporting data on the umbrella category of ‘Asian Australians’ have the potential to gauge racism directed towards a broad group vulnerable to discrimination, but may obscure the impact on particular subgroups, including those perceived as being of Chinese background. Finally, studies which focus exclusively on discrimination in face-to-face settings may obscure the impact of lockdowns and levels of other modalities—such as online or everyday racism.

## The Study

We analysed data from an online survey of Asian Australians living in Victoria, facilitated by the research firm Qualtrics and conducted between 3 May 2021 and 7 June 2021. This was after two periods of lockdown (31 March 2020 to 12 May 2020 and 9 July 2020 to 27 October 2020) and overlapped with the third (28 May 2021 to 10 June 2021). The Delta/B.1.617/ ‘Indian’ variant emerged at the mid-way point of the survey.

All participants were adults (18 years of age or older), self-identified as Asian Australian and had their primary residence in the state of Victoria. After screening measures were applied, a sample of 436 respondents was derived. No personal or identifying information was recorded. In accordance with the project’s ethics approval, small incentives were provided to participants by Qualtrics’ partner organisations. The sample was representative of key demographic indicators for Victoria. Nearly 71% of respondents lived in metro locations, 22% in regional locations and 6.8% in rural locations. Approximately 50% of participants identified as female, 48.9% as male, 0.7% preferred not to specify a gender, and 0.5% preferred descriptions such as ‘gender neutral’ or ‘they’. A total of 33 ethnicities and 37 primary languages were self-identified by participants. The largest groups were Indian (19.5%), Chinese (19.2%), Vietnamese (9.2%), Nepali (8%) and Sri Lankan (7.1%). Among the participants, 2.9% identified as Taiwanese, and 2.5% as Hong Kongese. 51.8% of respondents reported speaking a language other than English when at home (Mandarin 7.1%, Vietnamese 5.7%, Nepalese 5.2% and Hindi 5%). Most of the sample (71.4%) were first-generation immigrants, 19.5% were second-generation immigrants and the remainder were third-generation immigrants (4.6%). A total of 47 countries of birth were identified, including Australia (24%), India (17.2%), China (6%), Nepal (8%), Sri Lanka (5.9%) and the Philippines (5.9%). To gain more clarity in relation to modalities of racism, the survey asked respondents 15 questions about experiences of racism and discrimination associated with the pandemic, the forms such incidents took, and their impact on feelings of anxiety and sense of belonging. Respondents were asked to complete some questions twice, once with the prompt, ‘in the YEAR PRIOR to the COVID-19 pandemic’ and again with the prompt, ‘since the BEGINNING of the COVID-19 pandemic’. Responses for each question were recorded on a five-point, Likert type scale with the options of never, hardly ever, sometimes, often and very often and were scored between 0 (never) and 4 (very often). For the purpose of analysis, responses to these questions were then aggregated to create four measures adapted from previous Australian research. These were Direct Experience of Racism, Vicarious Experiences of Racism, Everyday Racism and Hypervigilance. The scores for each measure were derived by summing the score for each item in the measure.

### Direct Experiences of Racism

The Direct Experiences of Racism measure, adapted from Biddle et al. (2020), sought information on incidents of racism and discrimination directly experienced by participants in a range of settings. It presented participants with the question, ‘how often did you experience discrimination that you felt was connected to your racial or cultural background?’ at work, at school, TAFE or university, renting or buying property, interactions with the police or court system, seeking healthcare, shopping or dining at a restaurant, at a sporting event, on the street or public transport, online, at home or at a friend/family member’s home. This measure included in-person as well as online experiences. (Year prior—min = 0, max = 40, MN = 9.76, SD = 8.64. Beginning COVID—min = 0, max = 40, MN = 9.14, SD = 9.44. Alpha = 0.94).

### Vicarious Experiences of Racism

The Vicarious Experiences of Racism measure, adapted from Doery et al. (2020), sought information on observed rather than directly experienced incidents of racism and discrimination. However, in addition to asking about experiences online and experiences of family and friends we sought information about indirect racism ‘in person’. Respondents were presented with the prompt: ‘Witnessing acts of discrimination or racism can have a similar impact to experiencing them directly. We’re interested to know what you may have witnessed and where the incident(s) occurred (IN PERSON or ONLINE)’. This was followed by the question, ‘In the YEAR PRIOR [or BEGINNING of] to the COVID-19 pandemic, how often have the following things happened:’. Three follow-ups were provided in a Likert matrix for both ‘in person’ and ‘online’ sections. These were ‘I have witnessed people making jokes about others of my racial, ethnic or cultural group’, ‘I have witnessed people say rude or insulting things about another person’s racial, ethnic or cultural group’, and ‘My family or friends have experienced discrimination or unfair treatment because of their racial, ethnic or cultural background’. (Year prior—min = 0, max = 24, MN = 8.18, SD = 6.01. Beginning COVID—min = 0, max = 24, MN = 8.53, SD = 6.55. Alpha (in person) = 0.89, alpha (online) = 0.87).

### Everyday Racism

The Everyday Racism measure, adapted from the ‘Challenging Racism Project 2015–16 National Survey Report’ (Blair et al., 2017), sought information about forms of racially based hostility that are less explicit and typically more repetitive than overt acts of discrimination. Following Blair et al. (2017), it allows distinctions between racism in institutions, in everyday settings and ‘subtle racisms’ such as being treated disrespectfully, not being trusted or being called names (Blair et al., 2017, p. 11). In this study, respondents were asked ‘how often did you feel that because of your racial or cultural background…’ in relation to three prompts: ‘you were treated in a less respectful manner’, ‘people acted or behaved as if you were not to be trusted’, and ‘you were called names or similarly insulted’. (Year prior—min = 0, max = 12, MN = 3.15, SD = 2.84. Beginning COVID—min = 0, max = 12, MN = 3.29, SD = 3.06. Alpha = 0.89).

### Hypervigilance

In addition to these, we developed a Hypervigilance measure, adapted from Doery et al. (2020), to seek information on anxiety and stress associated with experiences of racism. Respondents were asked the question, ‘how often did you…’ in relation to four prompts: ‘Feel that you needed to prepare for possible insults from others before leaving home’, ‘Feel that you had to be careful about your appearance or way you presented yourself (for example, to avoid being harassed, or to receive adequate service at a shop/restaurant)’, ‘Feel that you needed to pay extra attention to what you said and how you said it’, and ‘Feel that it was best to avoid certain social situations and places’. (Pre-COVID—min = 0, max = 16, MN = 4.69, SD = 4.16. Beginning COVID—min = 0, max = 16, MN = 5.42, SD = 4.51. Alpha = 0.92).

### Sample Subgroups

To gain more specific insights into the category ‘Asian Australians’, we created two subgroups. To identify those likely to be perceived as of Chinese background, participants from East and Southeast Asian (ESE) backgrounds were aggregated, creating a group of participants with Chinese, Japanese, Hong Kongese, Taiwanese, Korean, Macanese, Okinawan, Mongolian, Vietnamese, Filipino, Malaysian, Singaporean, Indonesian, Thai, Cambodian, Burmese, Timorese and Bruneian cultural backgrounds. The second, ‘control’ group, aggregated participants with West, South and Central Asian (WSC) backgrounds, creating a group consisting of participants of Armenian, Georgian, Azerbaijani, Indian, Nepali, Sri Lankan, Pakistani, Bangladeshi, Bhutanese, Turkmen, Uzbek, Tajik, Kyrgyz and Kazakh cultural backgrounds. The ESE group comprised 250 (57.3% of the total sample) respondents. Eighty-three (43.2%) were female and 108 (44.8%) were male. A further 4 (1.6%) respondents either did not specify a gender or preferred other gender pronouns. Of these respondents, 82 (32.8%) were born in Australia while 168 (67.2%) were born overseas and 138 (55.2%) reported speaking English at home. The WSC group comprised 186 (42.6% of the total sample) respondents of which 105 (56.5%) identify as female and 80 (43%) identify as male. A further respondent did not specify a gender. Of these respondents, 23 (12.4%) were born in Australia while 163 (87.2%) were born overseas and 72 (38.7%) reported speaking English at home.

## Results

To address our first research question (Did people who were likely to be perceived as Chinese experience an increase in racism compared to other Asian Australians in Victoria during the pandemic?), we compared results for our four measures for the entire sample with results for two sub-groupings. Exploratory analysis was conducted using Tableau and R. Shapiro–Wilk tests were used to assess normality of sub-group distributions. This showed that distributions for all measures across both sub-groups were not normally distributed and demonstrated strong positive skews. As such, Wilcoxon signed rank tests were used to evaluate the differences between pre-COVID (prompted with ‘in the year PRIOR to the COVID-19 pandemic…’) and post-COVID (prompted with ‘since the BEGINNING of the COVID-19 pandemic…’) test scores for each measure. Effect sizes were calculated for statistically significant results (*p* < 0.05). Quantifying the results in terms of measures of magnitude, the effect size is reported to give an overall sense of how much participants were affected by the ‘experimental condition’—in this case, the COVID-19 pandemic. An *r* value that varies between 0.1 and 0.3 can be considered a small effect, 0.3–0.5 a moderate effect, and > 0.5 a large effect (Cohen, 1988). Further descriptive analysis of measure items was undertaken where a statistically significant result was detected.

Tables 1, 2 and 3 compare pre- and post-COVID test scores for our four measures for each of the sample groupings.

**Table 1:** Wilcoxon signed rank test results for whole sample

Measure	*N*	*MD* (pre-COVID)	*MD* (post-COVID)	*Z*-stat	*p* (*a* = 0.05)	*r* (abs)
Direct Racism	436	9.00	6.00	3.70	< 0.00*	0.18
Vicarious Racism	436	8.00	8.00	− 1.61	0.11	–
Everyday Racism	436	3.00	3.00	− 0.93	0.35	–
Hypervigilance	436	4.00	5.00	− 5.52	< 0.00*	0.26

*Indicates statistically significant result

**Table 2:** Wilcoxon signed rank test results for respondents with an East and Southeast Asian cultural background

Measure	*N*	*MD* (pre-COVID)	*MD* (post-COVID)	*Z*-stat	*p* (*a* = 0.05)	*r*
Direct Racism	250	6.00	5.00	1.70	0.08	–
Vicarious Racism	250	7.00	8.00	− 3.08	< 0.00*	0.19
Everyday Racism	250	2.00	3.00	− 3.52	< 0.00*	0.22
Hypervigilance	250	4.00	5.00	− 6.76	< 0.00*	0.43

*Indicates statistically significant result

**Table 3:** Wilcoxon signed rank test results for respondents with a South, West and Central Asian cultural background

Measure	*N*	*MD* (pre-COVID)	*MD* (post-COVID)	*Z*-stat	*p* (*a* = 0.05)	*r*
Direct Racism	186	11.00	8.50	3.63	< 0.00*	0.27
Vicarious Racism	186	9.00	8.50	1.19	0.23	–
Everyday Racism	186	3.00	3.00	2.80	< 0.00*	0.21
Hypervigilance	186	5.50	5.00	0.07	0.94	–

*Indicates statistically significant result

Table 1, presenting results for the whole sample, shows either a small or no increase for Vicarious Racism, Everyday Racism and Hypervigilance. There is a small decrease for Direct Racism between pre- and post-COVID scores. However, Table 2 shows that for ESE respondents there were statistically significant increases in Vicarious Racism, Everyday Racism and Hypervigilance. The impact of the measures of Vicarious Racism (*r* = 0.19) and Everyday Racism (*r* = 0.22) were small, while the impact of Hypervigilance was moderate (*r* = 0.43). For the ESE group, however, there was no increase in Direct Racism. In Table 3, the results for the WSC group show a statistically significant decrease in scores for Direct Racism, and Everyday Racism showed no change.

These findings support an affirmative, but qualified, answer to our first research question: people who were likely to be perceived as Chinese did experience an increase in racism compared to WSC group, in three measures: Vicarious Racism, Everyday Racism and Hypervigilance. There was no statistically significant increase for Direct Racism.

To address our second research question, ‘What were the predominant modes in which this racism was experienced?’, we compared responses for online and in-person experiences in our Vicarious Racism measure for the ESE group (Table 4) and used descriptive data analysis for Vicarious Racism, Everyday Racism and Hypervigilance (Tables 5, 6 and 7). Table 4 shows that there was no difference pre- and post-COVID in relation to in-person Vicarious Racism. However, online Vicarious Experiences of Racism increased, with a borderline moderate effect (*r* = 0.28).

**Table 4:** Wilcoxon signed rank test results for Vicarious Racism—online and in-person. Respondents with an East and Southeast Asian cultural background

Measure	*N*	*MD* (pre-COVID)	*MD* (post-COVID)	*Z*-stat	*p* (*a* = 0.05)	*r* (abs)
Online	250	4.00	5.00	− 4.48	< 0.00*	0.28
In-person	250	3.00	3.00	− 0.54	0.59	–

*Indicates statistically significant result

**Table 5:** Descriptive stats for online Vicarious Experiences of Racism. Respondents with an East and Southeast Asian cultural background

Question	Pre-COVID (% ‘often’ and ‘very often’)	Post-COVID (% ‘often’ and ‘very often’)	% change
Witnessing jokes	17.20	29.60	72.09 (increase)
Witnessing rude or insulting things	18.80	30.40	61.70 (increase)

**Table 6:** Descriptive stats for Everyday Racism. Respondents with an East and Southeast Asian cultural background

Question	Pre-COVID (% ‘often’ and ‘very often’)	Post-COVID (% ‘often’ and ‘very often’)	% change
Treated in a less respectful manner	7.60	12.40	63.15 (increase)
Called names and similar insults	8.00	10.40	30 (increase)
Not trusted	6	10.4	73.33 (increase)

**Table 7:** Descriptive stats for Hypervigilance. Respondents with an East and Southeast Asian cultural background

Question	Pre-COVID (% ‘often’ and ‘very often’)	Post-COVID (% ‘often’ and ‘very often’)	% change
Prepare for possible insults	8.49	15.69	80.95 (increase)
Appearance and presentation require careful consideration	10	16	60 (increase)
Pay extra attention to what they said and how they said it	10.40	19.95	92.30 (increase)

Descriptive analysis of our data on online Vicarious Racism (Table 5) identified jokes and instances of rude or insulting content as the most significant. When asked about witnessing jokes being made about others of a respondents’ cultural or ethnic background the year prior to the pandemic, 17.20% of ESE respondents selecting ‘often’ or ‘very often’ increasing by 72.09 to 29.60% during the pandemic. When asked about witnessing people saying rude or insulting things about another’s cultural or ethnic background online, 18.80% of ESE respondents selected ‘often’ or ‘very often’ for the year prior to the pandemic, increasing by 61.70 to 30.40% when asked to consider the same question during the pandemic.

On the Everyday Racism measure (Table 6), respondents reported a 63.15% rise in being treated in a less respectful manner ‘often’ or ‘very often’. There was a 30% increase reported in relation to being called names or facing a similar kind of assault ‘often’ or ‘very often’. An increase was also reported in incidents of not being trusted during the pandemic, with 10.4% of participants answering ‘often’ or ‘very often’ for the year during the pandemic, up 73.3% from the year prior.

Table 2 shows a significant increase in levels of anxiety among our participants during the pandemic. The Hypervigilance measure recorded the largest increase of all measures and the highest impact score (*r* = 43). Descriptive analysis of Hypervigilance (Table 7) for ESE respondents shows significant increases on all questions, but the greatest concerns were in relation to appearance and language. An increase of 80.95% was recorded for respondents feeling that they needed to prepare for possible insults from others before leaving home ‘often’ or ‘very often’. There was a 60% increase in participants reporting that appearance and presentation required careful consideration before leaving home ‘often’ or ‘very often’. Significantly, the number of ESE respondents reporting ‘often’ or ‘very often’ having to pay extra attention to what they said and how they said it almost doubled, from 10.4% before the pandemic, to 20%, during the pandemic.

These findings provide a preliminary response to our second research question. The ESE group reported increases in the experience of racism within the following modes: Vicarious Experiences of Racism online, Everyday Racism and Hypervigilance.

## Discussion

As research on the shadow pandemic has grown, the variability of its scale, modalities and impact across national contexts has increasingly come into focus (Delvin & Connaughton, 2020). In this paper, we have sought more clarity about the experiences of racism in Australia by focusing on a specific context (the Australian state of Victoria), by distinguishing between people likely to be perceived as Chinese and other Asian Australians, and by examining in-person, online and everyday modalities of racism. In doing this, we have sought to shed light on the seemingly contradictory results of previous Australian research.

It is not possible to draw definitive conclusions about impact of different circumstances in different state jurisdictions on experiences of racism during the pandemic without comparative studies involving other states. However, our focus on the hardest hit state in the country has provided data and conclusions that differ from those of national studies, throwing up the possibility that the circumstances that prevailed in Victoria may have had a bearing on the modes and levels of racism experienced. Further research will be required to confirm this possibility.

Our study suggests that, in Victoria, racism did not affect all Asian Australians equally. Previous Australian research has both recognised that Chinese Australians are likely to be the primary target of COVID-19 related racism (Biddle et al., 2020, p. 2; Kamp et al., 2022, p. 1) and noted that COVID-19-related racism and discrimination has cut across various minoritised groups, including non-Asians (Elias et al., 2021). For the most part, however, previous analyses have not separated Asian Australians who were likely to be perceived as Chinese from the broader category of Asian Australians. By making this distinction, our analysis has shown while Asian Australians considered as a whole did not report an increase in racism, this group did.

This finding is in line with international research showing a spike in racism directed towards people likely to be perceived as Chinese (Croucher et al., 2020; Gover et al., 2020; European Union Agency for Fundamental Rights, 2020; Haft & Zhou, 2021; Pan et al., 2020; Zhou et al., 2021). However, it paints a different picture to the studies which pointed to a shift in the primary target of pandemic-related racism within Asian communities over the course of the pandemic. The most notable shift concerned reports of racism targeting South Asian communities, especially in the USA and Canada, at the time of the Delta/B.1.617/ variant outbreak in India (Kamp et al., 2022). In our study, despite the Delta outbreak occurring mid-way through our survey, we found no evidence of a targeting of the Indian community. Victorians of Indian background comprised a significant proportion (45.5%) of the WSC group in our study. Yet this group reported a decrease in scores for Direct Racism and no change for Everyday Racism, in contrast with the increase recorded on both measures for the ESE group.

Further research is required to explore the factors underlying this contrast with other White settler nations such as such the USA and Canada. One factor that may have played a role in Australia is the country’s very different histories of anti-Chinese and anti-Indian racism. Chinese Australians have had a long presence in the country, including during its period of racially based immigration restrictions and ‘White Australia’ policies which continued until the mid-twentieth century (Fitzgerald, 2007). In contrast, the Indian Australian community has achieved critical mass only in the last several decades. While there have been significant upsurges of racism targeting Indians (Dunn et al., 2011; Mason, 2012), and the federal government’s temporary stay on Australian citizens returning from India during the Delta wave were widely denounced as racist (BBC, 2021), research has pointed to a marked discrepancy in levels of negative sentiment experienced by the two communities, with one survey reporting that 27% of Australians reported negative or very negative attitudes towards people from India, compared with 43% towards those from China (Scanlon Foundation, 2021, p. 13).

The third set of observations that can be made on the basis of our data concern the modalities of racism experienced by Victorians likely to be perceived as Chinese. Like previous Australian studies, we recorded no increase in in-person experiences of racism (either directly or vicariously) for this group. The consistency in findings for in-person racism, considered alongside more variable findings in relation to other modalities, lends weight to suggestions that the absence of an increase in in-person racism at least in part stemmed from a reduction in opportunities during the pandemic for encounters in the settings in which they typically occur. However, our findings in relation to Everyday Racism invite a more nuanced interpretation of this finding.

In contrast to the absence of an increase in in-person racism, we found a marked increase in Everyday Racism for the ESE group. A direct comparison with previous Australian studies on this point is not possible. While they gathered data on the component items of our Everyday Racism measure—particularly respect and trust—they utilised them in different ways. For example, Kassam and Hsu (2021, 2022) gathered data on ‘trust in governments’ but not in fellow Australians. Biddle et al. (2020) sought data on levels of interpersonal trust in other Australians but saw it as a measure of social cohesion rather than as a measure of racism and discrimination. In contrast, our findings suggest that respondents felt that not being trusted or respected was a common form of racism of significant concern. Our findings also suggest an alternative interpretation of the discrepancy between the in-person and everyday measures. Our Everyday racism measure did not specify a setting and thus cut across the distinction between in-person and online settings. While it is therefore not possible to know where the incidents of everyday racism reported by our respondents took place, it is probable that at least some took place in in-person settings. To the extent that this was the case, we can speculate that some respondents did not consider their experiences of everyday forms of in-person racism as ‘racism’ or ‘discrimination’ as such.

Similarly, we found a significant increase in vicarious online racism for the ESE group. This finding was not surprising. A large body of research predating the pandemic documented the growing salience of online fora as a major site of race-related hostility and hate (Bliuc et al., 2018; Klein, 2017). At the same time, reports of a significant increase in time spent online during the pandemic (Spanos, 2020) suggest that opportunities for racism in this setting may have grown while those in face-to-face settings declined. It also accords with Australian (Doery et al., 2020) and international research (Croucher et al., 2020; European Union Agency for Fundamental Rights, 2020) which has pointed to a rise in online racism during the pandemic, including one robust study which found a marked increase in anti-Asian sentiment on Twitter following the emergence of COVID-19 in the USA (Nguyen et al., 2020). However, it is seemingly at odds with the findings from Kamp et al. (2022). This latter study found that while the pandemic was a predominant theme in instances of online racism—and therefore a cause for concern—there was no increase in level of incidents of online racism targeting Asian Australians.

However, our research design helps to shed light on these disparate findings. By comparing data for the ESE group, the WSC group and the sample as a whole, we have shown that differential rates of reported incidences of racism among Asian Australians can obscure an increase in incidents of racism targeting those likely to be perceived as of Chinese background. Our data for the sample as a whole shows, like Kamp et al. (2022), no increase in vicarious online racism. Further analysis, however, reveals an increase for the ESE group that was offset by a decrease in the WSC group.

## Conclusion

Our study has shed light on the seemingly contradictory picture painted in previous research on pandemic-related racism in Australia, of an increase in COVID-19-related racism and xenophobia without any overall increase in experiences of racism and discrimination. We have shown that, in Victoria, the hardest-hit state in Australia, Asian Australians likely to be perceived as of Chinese background experienced an increase in levels of racism. We also have shown that this occurred within the modalities of online and ‘everyday’ racism. In doing this, we have shown that failing to differentiate between different subgroups of Asian Australians, or to canvas a spectrum of racisms may have tended to obscure this more targeted experience in previous research.

In delving into the nuances of the impact of the pandemic on Asian Australians, we have highlighted issues that are important for ongoing efforts to understand racism in Australia. While canvassing modalities of racism beyond those which occur in in-person settings was particularly important during the period of lockdowns and other social distancing measures, our study has highlighted the importance of online and everyday racism for research on racism more broadly. The salience of online settings as a platform for racism is a global trend with little sign of abating. At the same time, the issue of ‘everyday’ racism is acquiring greater prominence in Australia. Some commentators have identified it as more common and more pernicious than the crude expressions which attract the most media attention (Aly, 2013). Reflecting this salience, the Australian Human Rights Commission relaunch of its *Racism. It Stops With Me* campaign includes a focus on subtle forms of racism (2022). Overemphasis on the notion of everyday forms of racism has been criticised for neglecting structural or institutional racism (Lentin, 2017). However, those arguing for the significance of everyday racism have stressed its embeddedness in institutional and cultural frameworks, as well as drawn attention to its potentially far-reaching impact on those it targets (Kwansah-Aidoo & Mapedzahama, 2018; Walton et al., 2013; Ballantyne & Podkalicka, 2020). Above all, its relevance is underscored by the changes that have been wrought in Australia’s diversity landscape by the country’s shift, since the 1990s, to high-skilled, predominantly Asian, migrant recruitment. For new skilled migrants—among whom Chinese and Indian Australians are prominent—a sense of belonging based on feeling trusted and respected is often more important than addressing economic disadvantage.

Growing evidence of a shadow pandemic of heightened racism and xenophobia has been a concerning feature of the emergence of COVID-19. While revealing an increase in racism directed at Australians likely to be perceived as Chinese, our study, in line with previous research, also suggests that racism sparked by the COVID-19 pandemic in Australia was relatively contained. It shows that the majority of Asian Australians in our sample, including those likely to be perceived as of Chinese background, did not experience elevated rates of racism. This conclusion is encouraging. However, our study, like previous research, has also highlighted a concerning level of pre-pandemic racism. This suggests that the COVID-19 pandemic has been an opportunity for longstanding currents of anti-Asian racism to surface (Wu et al., 2021), and that further investigation of the drivers and nature of those currents remain urgent.
